# Successful Non-surgical Management of an Infected Femoral Pseudoaneurysm and Metastatic Septic Arthritis Following Percutaneous Coronary Intervention in a Hemodialysis Patient

**DOI:** 10.7759/cureus.85586

**Published:** 2025-06-09

**Authors:** Hiroki Ito, Keiji Matsumoto, Takuo Hirose, Takefumi Mori

**Affiliations:** 1 Division of Nephrology and Hypertension, Tohoku Medical and Pharmaceutical University, Sendai, JPN

**Keywords:** hemodialysis, infected pseudoaneurysm, mrsa, percutaneous coronary intervention, septic arthritis

## Abstract

Infectious complications following percutaneous coronary intervention (PCI) are uncommon but potentially devastating, particularly in hemodialysis patients who experience immunosuppression and elevated methicillin-resistant *Staphylococcus aureus* (MRSA) colonization rates. We present the case of a 68-year-old woman on hemodialysis who developed an MRSA-infected femoral pseudoaneurysm and contralateral septic shoulder arthritis five days after femoral-access PCI. Despite both conditions traditionally warranting surgical intervention, conservative management with six weeks of targeted intravenous vancomycin therapy achieved complete resolution. This case demonstrates the rapid progression potential of severe infections in immunocompromised hemodialysis patients and highlights the efficacy of antibiotic therapy alone in carefully selected high-surgical-risk patients. Success factors included prompt clinical response, contained infection, and comprehensive clinical and radiological monitoring.

## Introduction

Infectious complications following percutaneous coronary intervention (PCI) occur infrequently but carry substantial morbidity and mortality, especially among maintenance hemodialysis patients [1]. These patients face heightened susceptibility due to several factors: uremia-induced immunosuppression, frequent vascular access procedures, and elevated rates of methicillin-resistant *Staphylococcus aureus* (MRSA) colonization [1,2].

Uremia-associated immune dysfunction manifests as impaired neutrophil function, deficient T-cell responses, and dysregulated cytokine production [2]. Common comorbidities in this population, such as diabetes mellitus and established cardiovascular disease, further increase infection risk [3].

Iatrogenic femoral pseudoaneurysms occur in 0.2%-0.5% of diagnostic angiograms and up to 8% of PCIs [4-6]. Risk factors include larger sheath size, advanced age, elevated body mass index, female gender, hypertension, concurrent anticoagulation, and arterial calcification [7]. Prolonged femoral sheath retention (>24 hours) represents a particularly significant risk factor for post-PCI infections [1].

While surgical intervention has traditionally been the standard approach for infected pseudoaneurysms and septic arthritis [8], limited emerging evidence suggests that conservative management may be viable in carefully selected cases, particularly for patients at high surgical risk [9]. Small, asymptomatic, non-infected pseudoaneurysms (<2 cm) can be successfully managed with serial ultrasonographic monitoring [10]. However, for infected pseudoaneurysms, percutaneous thrombin injection, commonly used for non-infected lesions, is generally contraindicated due to infection dissemination risk [11,12].

As the global dialysis population continues to age, with median age in developed countries now exceeding 65 years [13], alternative management strategies for high-surgical-risk patients become increasingly relevant. Recent registry data show that over 40% of incident dialysis patients are now over 70 years of age, with multiple comorbidities that significantly elevate perioperative risk [14]. The mortality rate for emergent vascular surgery in dialysis patients ranges from 10% to 30%, approximately two to four times higher than in non-dialysis patients undergoing similar procedures [15,16].

We describe a case of rapidly evolving MRSA-infected femoral pseudoaneurysm with concurrent metastatic septic arthritis following PCI in a hemodialysis patient that uniquely resolved with conservative antimicrobial therapy alone. This case contributes to the limited but growing evidence supporting selective non-surgical management in specific high-risk scenarios.

## Case presentation

A 68-year-old woman with a 20-year history of type 2 diabetes mellitus and six years of maintenance hemodialysis presented for coronary intervention. Initial coronary angiography revealed two-vessel disease: 75% stenosis in segment #7 and 90% stenosis in segment #11. She underwent percutaneous intervention via right femoral approach for the segment #7 lesion. Her medications included dual antiplatelet therapy (aspirin and prasugrel), standard antihypertensive agents, and antidiabetic medications. Glycemic control was adequate with glycoalbumin levels of 18%-19%. The procedure employed standard sterile technique with a 6-French sheath.

Five days post-procedure during routine hemodialysis, she developed fever (38.9°C), right groin pain, and left shoulder pain. Physical examination showed blood pressure at 142/85 mmHg, heart rate at 98 beats/minute, respiratory rate at 18 breaths/minute, and oxygen saturation 98% on ambient air. Key findings included an egg-sized, tender induration in the right groin and left shoulder swelling with limited range of motion. Laboratory tests revealed marked inflammation with leukocytosis (white blood cell count 16,100/μL), and elevated C-reactive protein (29.22 mg/dL) and procalcitonin (6.99 ng/mL) levels (Table 1).

**Table 1:** Blood analysis on admission AST, aspartate aminotransferase; ALT, alanine aminotransferase; γ-GTP, gamma glutamyl transpeptidase; HbA1c, hemoglobin A1c; eGFR, estimated glomerular filtration rate; LDL, low-density lipoprotein; CRP, C-reactive protein; BNP, brain natriuretic peptide

Test	Result	Unit	General reference value
Complete blood count			
White blood cell	16,100	/μL	4,000-9,000/μL
Neutrophils	88.2	%	40%-75%
Hemoglobin	9.0	g/dL	12.0-15.5 g/dL
Red blood cell	2.92	×10⁶/μL	4.0-5.0 × 10⁶/μL
Hematocrit	27.8	%	36%-44%
Platelets	33.4	×10⁴/μL	15.0-45.0 × 10⁴/μL
Biochemistry			
Total protein	6.3	g/dL	6.0-8.3 g/dL
Albumin	2.7	g/dL	3.5-5.5 g/dL
AST	23	U/L	5-40 U/L
ALT	16	U/L	7-42 U/L
Total bilirubin	0.63	mg/dL	0.1-1.2 mg/dL
γ-GTP	16	U/L	5-36 U/L
HbA1c	6.8	%	<5.7%
BUN	25	mg/dL	7-20 mg/dL
Creatinine	4.77	mg/dL	0.6-0.9 mg/dL
eGFR	7.7	mL/min/1.73 m²	≥90 mL/min/1.73 m²
Glucose (random)	145	mg/dL	≤180 mg/dL (random)
Uric acid	2.7	mg/dL	2.4-6.0 mg/dL
Sodium	139	mEq/L	135-145 mEq/L
Potassium	5.0	mEq/L	3.5-5.3 mEq/L
Chloride	98	mEq/L	98-107 mEq/L
Calcium, total	9.4	mg/dL	8.6-10.3 mg/dL
Phosphorus	4.1	mg/dL	2.5-4.5 mg/dL
Iron	16	μg/dL	50-170 μg/dL
Ferritin	535	ng/mL	10-200 ng/mL
Total cholesterol	170	mg/dL	<200 mg/dL
Triglycerides	201	mg/dL	<150 mg/dL
LDL cholesterol	92	mg/dL	<140 mg/dL
CRP	29.22	mg/dL	<1.0 mg/dL
Procalcitonin	6.99	ng/mL	<0.1 ng/mL
BNP	396.2	pg/mL	<100 pg/mL

**Figure 1:**
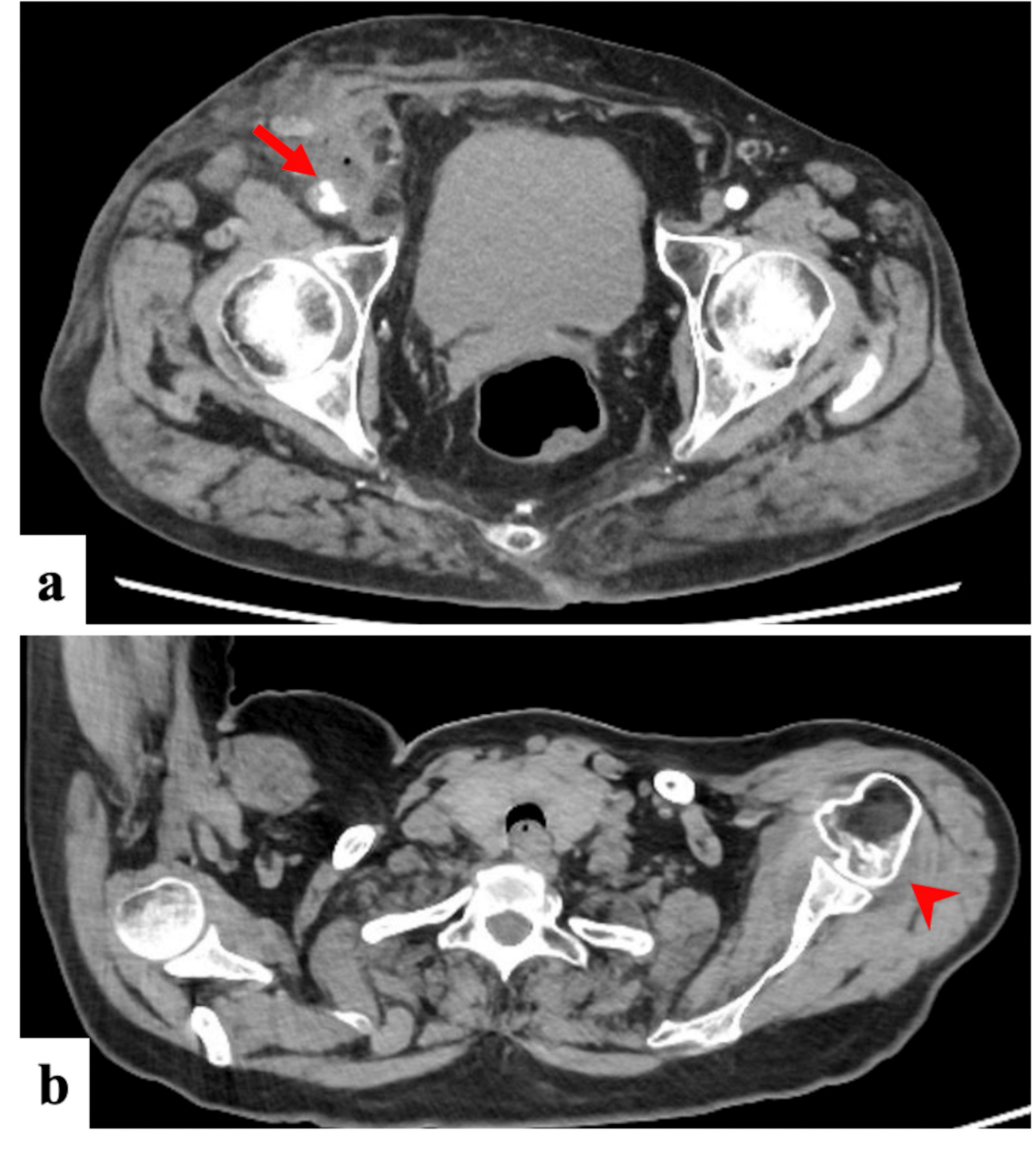
Initial computed tomography scans: infected pseudoaneurysm and contralateral septic arthritis (a) Contrast-enhanced computed tomography imaging of the right groin showing a 5-mm protrusion (arrow) on the ventral side of the right femoral artery with surrounding inflammatory changes including increased fat tissue density, internal gas formation, and regional lymphadenopathy, consistent with infected pseudoaneurysm formation. (b) Non-contrast computed tomography of the left shoulder demonstrating a low-density area around the left subscapularis muscle (arrowhead) with extension into the glenohumeral joint space, suggesting synovial fluid accumulation and septic arthritis.

Computed tomography (CT) showed a 5-mm protrusion on the ventral aspect of the right femoral artery with increased surrounding fat tissue density, gas formation, and mild lymphadenopathy, suggesting cellulitis/abscess and pseudoaneurysm formation (Figure 1a). CT also revealed a hypoattenuating area surrounding the left subscapularis muscle extending into the shoulder joint, indicating synovial effusion and arthritis (Figure 1b). Ultrasonography of the right groin demonstrated poorly defined femoral artery architecture with adjacent fluid collection. Echocardiography showed a mildly reduced ejection fraction (51%) without valvular abnormalities. Left shoulder joint aspiration yielded fluid with a markedly elevated cell count (62,650/μL) without turbidity or crystals (Table 2).

**Table 2:** Joint fluid analysis

Test	Result	Unit	General reference value
Number of cells	62,650	/μL	<200/μL
Neutrophils	91.0	%	<25%
Monocytes	6.0	%	2%-48%
Basophils	3.0	%	<2%
Monosodium urate crystals	(-)		Absent
Calcium pyrophosphate dihydrate crystals	(-)		Absent

Blood cultures, synovial fluid, and groin exudate all yielded methicillin-resistant *Staphylococcus aureus* with identical susceptibility patterns, confirming infected pseudoaneurysm and left shoulder septic arthritis. We systematically excluded infective endocarditis: physical examination showed no characteristic findings (no Roth spots, Osler's nodes, Janeway lesions, splinter hemorrhages, or embolic infarctions), fundoscopy revealed no septic emboli, and transesophageal echocardiography showed no vegetations.

Initial empiric therapy included vancomycin (a 1 g loading dose, and then 0.5 g post-dialysis) plus meropenem (0.5 g/day). After MRSA identification, treatment was narrowed to vancomycin monotherapy. Serial monitoring confirmed therapeutic vancomycin levels (a pre-hemodialysis target of 15-20 μg/mL, and post-hemodialysis target of 5-10 μg/mL), allowing continuation of the initial dosing regimen. The right groin underwent incision and drainage.

**Figure 2:**
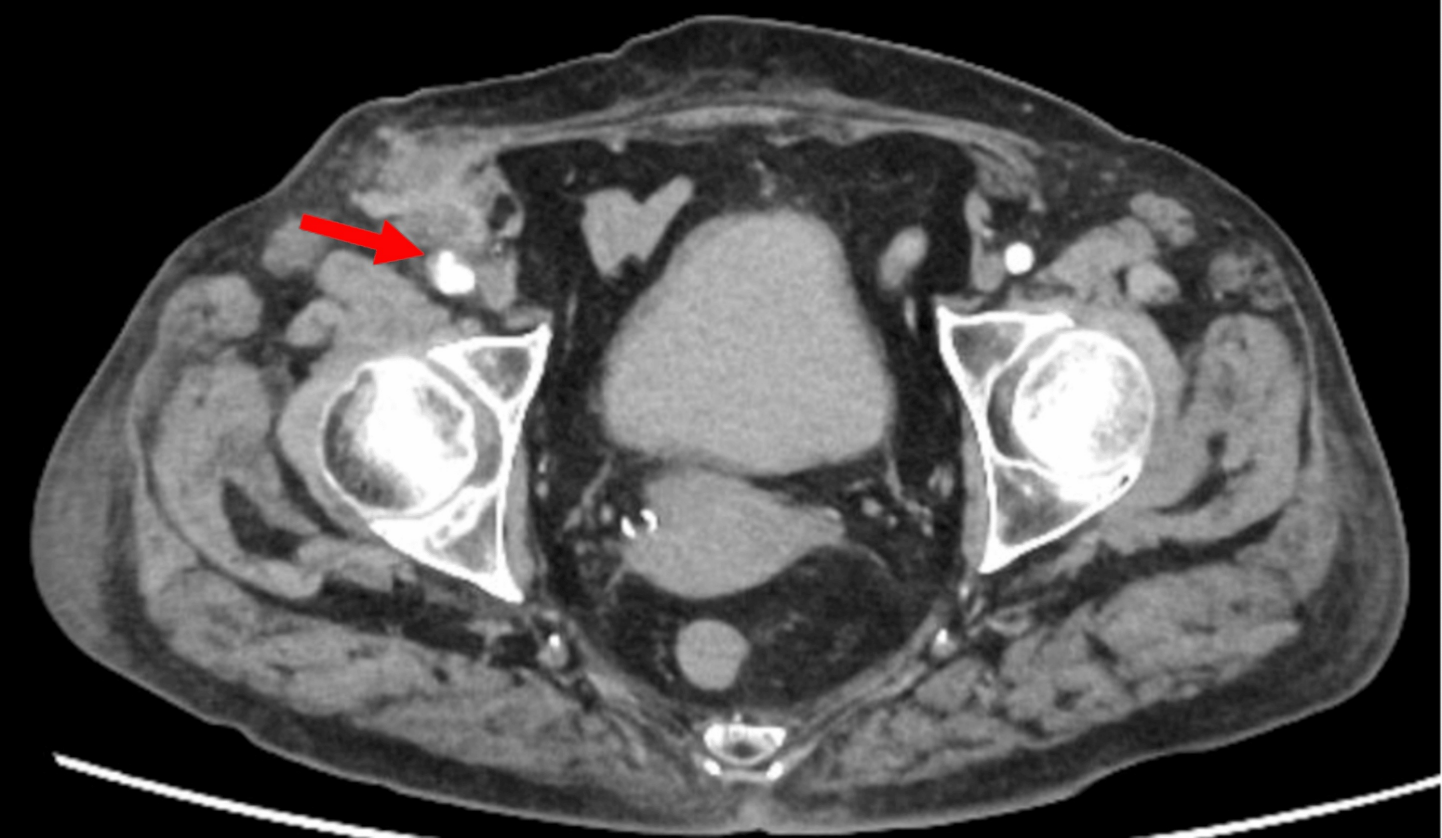
Follow-up computed tomography scan: femoral pseudoaneurysm during conservative management Follow-up contrast-enhanced computed tomography imaging showing the femoral pseudoaneurysm with a slight decrease in size and no evidence of expansion or impending rupture (arrow), supporting the decision to continue conservative management.

Despite conventional surgical indications for both conditions, we pursued conservative management with careful monitoring due to the patient's elevated surgical risk associated with end-stage kidney disease (including potential hemorrhagic complications, impaired wound healing, and vascular access compromise). Serial imaging showed stability of the infected pseudoaneurysm without progression (Figure 2). Shoulder symptoms improved steadily, and magnetic resonance imaging showed no worsening of joint effusion or destructive changes. The patient demonstrated prompt clinical improvement with rapid fever resolution. Laboratory markers improved substantially by hospital day 10 (leukocyte count, 8,000/μL; C-reactive protein, 1.83 mg/dL), supporting continued conservative management.

**Figure 3:**
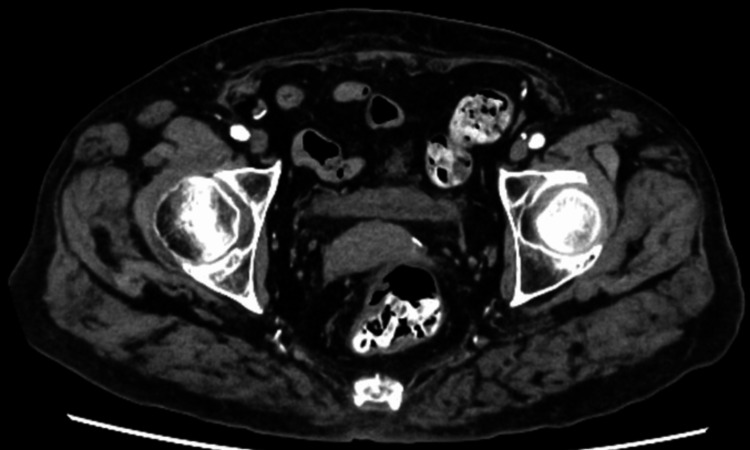
Final follow-up computed tomography scan: resolution of the femoral pseudoaneurysm Final follow-up contrast-enhanced computed tomography imaging showing resolution of the femoral pseudoaneurysm with no evidence of residual infection or inflammation.

Serial imaging demonstrated gradual pseudoaneurysm resolution without expansion or impending rupture. By hospital day 28, inflammatory markers approached normal ranges (leukocyte count, 5,200/μL; C-reactive protein, 0.21 mg/dL) and normalized completely at six weeks (leukocyte count, 5,800/μL; C-reactive protein, 0.06 mg/dL). After antibiotic discontinuation, inflammatory markers remained normal, and CT imaging confirmed complete resolution of the infected pseudoaneurysm (Figure 3) with complete resolution of shoulder symptoms. Furthermore, the patient has remained free of any recurrence of inflammation, septic arthritis, or infected pseudoaneurysm for two years following treatment.

## Discussion

This case offers pivotal insights into managing severe post-PCI infectious complications in hemodialysis patients. First, it underscores the rapid progression of such complications in this immunocompromised group. The simultaneous MRSA-infected femoral pseudoaneurysm and metastatic septic arthritis developing within five days post-PCI exemplifies this virulence [1]. This accelerated timeline from localized infection to systemic involvement aligns with previous reports in dialysis patients [17]. Hemodialysis patients have markedly higher MRSA colonization (45%-60%) [18] and *Staphylococcus aureus* bacteremia rates (up to 46.9 times more frequent) [19] than the general population. Our patient exhibited multiple risk factors for bacteremia, including advanced age, diabetes, and hypoalbuminemia [20], likely contributing to the aggressive course and highlighting the need for vigilant post-procedural surveillance. The increasing prevalence of atherosclerosis risk factors and immunosuppression may further elevate the incidence of these infections [21].

Second, this case highlights the potential efficacy of conservative antimicrobial therapy for conditions traditionally mandating surgery [8], such as infected pseudoaneurysms and septic arthritis. Infected pseudoaneurysms, common at the femoral artery [22], are serious complications resulting from microbial vessel wall degradation [23]. Surgical intervention is typically indicated for overt infection, rupture, ischemia, compressive symptoms, skin necrosis, large aneurysm size, or failure of other methods [24]. Indeed, the literature on infected pseudoaneurysms in hemodialysis patients overwhelmingly reports surgical management, often involving excision, ligation, and/or bypass [25-28]. For instance, series by Sulistyaningsih et al. [26] and Behera et al. [28] documented exclusively surgical management in their cohorts of hemodialysis patients (4 cases) and general patients with infected pseudoaneurysms (43 cases), respectively. Individual case reports also describe surgical interventions for MRSA-infected pseudoaneurysms in this population [25,27]. Consequently, the complete resolution of an infected pseudoaneurysm with antibiotic monotherapy, as in our patient, is noteworthy [9], especially given the scarcity of supporting data for such an approach. While endovascular covered stents are an option for pseudoaneurysms in high-risk patients, their use in infected fields is controversial [29,30]. Our case suggests that antibiotics alone may suffice in specific scenarios (e.g., a small lesion, susceptible organism, rapid clinical response), contrasting with the prevailing surgical paradigm.

Management of septic arthritis in hemodialysis patients also frequently involves surgical debridement, with reported rates varying from 50% to nearly 100% [31-33]. While some series report universal surgical intervention alongside antibiotics [32], others indicate that surgery was necessary in only about half of the cases, particularly when blood cultures were positive (25% requiring surgery vs. 57.5% with negative blood cultures) [31]. This latter finding aligns with our bacteremic patient who responded to antibiotics. Another series found that 25% of dialysis patients with septic arthritis responded to antibiotics alone, characterized by early diagnosis, prompt appropriate antibiotic initiation, and no joint destruction on imaging [34]. Although prompt joint drainage is crucial, the optimal modality (arthrocentesis vs. surgery) and precise indications for debridement versus aspiration in dialysis patients remain debated [35], suggesting a need for individualized approaches and further research into long-term functional outcomes [31-33,35]. Our patient’s complete resolution of septic arthritis with vancomycin monotherapy, alongside the pseudoaneurysm, further supports the idea that a conservative, antibiotic-focused approach can succeed under specific circumstances.

Several factors likely contributed to the successful conservative outcome in our patient. These included the small initial pseudoaneurysm size (5 mm), which may favor endogenous thrombosis with reduced bacterial burden (non-infected pseudoaneurysms <10 mm show higher spontaneous resolution rates [36]). Crucially, there was a prompt and significant clinical and laboratory response to appropriate antibiotic therapy, with rapid fever resolution and a marked decline in inflammatory markers (e.g., >50% C-reactive protein reduction by day 10), which was a positive predictor for conservative success [37]. Optimal vancomycin dosing, guided by pharmacokinetic monitoring to maintain therapeutic levels above the MRSA minimum inhibitory concentration, was essential. Vancomycin's pharmacokinetics in hemodialysis patients can allow for sustained therapeutic levels with post-dialysis dosing [38]. Radiological evidence confirmed infection containment and subsequent pseudoaneurysm regression without signs of rupture, and the septic arthritis showed limited effusion without destructive changes [39]. Close multidisciplinary monitoring allowed for early detection of potential deterioration.

This approach was also influenced by the patient's high surgical risk associated with end-stage kidney disease [13] and frailty (frailty index 0.38). Elderly and frail hemodialysis patients [40], a growing demographic [13,41], present significant surgical challenges due to comorbidities [42]. Indeed, the frailty prevalence in the dialysis population ranges from 30% to 70% depending on assessment tools [43], representing a substantial population that might benefit from carefully selected non-surgical options. These patients face substantially higher perioperative mortality (e.g., three to four times higher for non-cardiac surgery in frail vs. non-frail dialysis patients [44], and up to 20% for infected peripheral pseudoaneurysm repair [21]). Thus, a well-monitored conservative strategy offers a potential alternative to high-risk surgery in carefully selected individuals, potentially avoiding significant morbidity and mortality.

The six-week duration of intravenous vancomycin aligned with general recommendations for prosthetic vascular graft or other endovascular infections, which typically advise six to eight weeks, or longer if persistent infection is evident [45,46]. Our patient's sustained normalization of inflammatory markers and complete radiological resolution supported the adequacy of this regimen. This successful non-surgical outcome for an infected pseudoaneurysm contrasts with the predominantly surgical management reported in the literature [25-28], which often involves ligation and/or bypass grafting. Similarly, combined medical and surgical therapy remains the standard for septic arthritis in this population [31-33]. While non-infected pseudoaneurysms are often managed with minimally invasive techniques like ultrasound-guided thrombin injection [10,47] or coil embolization [48], these are generally contraindicated in the presence of infection [11,12] due to dissemination risk or potential for failure [11,12,16].

Based on our experience and a literature review, we propose that considering conservative management for infected pseudoaneurysms in dialysis patients may be viable under specific circumstances. Essential criteria include the following: small aneurysm size (ideally <10 mm) without expansion, absence of hemodynamic instability or impending rupture, prompt clinical (e.g., defervescence within 48-72 hours) and laboratory response (significant inflammatory marker reduction within 7-10 days) to appropriate antimicrobial therapy, absence of extensive tissue necrosis or uncontained infection, high surgical risk due to comorbidities/frailty, and the capability for intensive clinical and radiological monitoring with readily available vascular surgical expertise for rescue if needed. While surgery remains the definitive standard of care for most infected pseudoaneurysms and septic arthritis, our case provides proof-of-concept that a non-operative pathway can succeed under these specific conditions. Close multidisciplinary collaboration (infectious disease, vascular surgery, radiology) is a prerequisite, maintaining a low threshold for surgical intervention should deterioration occur.

However, several limitations warrant acknowledgment. This is a single case report, thus limiting the generalizability of our findings. The pseudoaneurysm's relatively small initial size (5 mm) might have significantly contributed to the success of conservative management, as smaller lesions (<2 cm) have a higher propensity for spontaneous thrombosis, even if less predictable in relation to infection [10]. Furthermore, the rapid and positive clinical response observed may not be universal, underscoring the need for rigorous patient selection criteria for non-operative strategies.

## Conclusions

In conclusion, our case demonstrates that exclusive antimicrobial therapy, when meticulously monitored, can achieve successful resolution of both an infected femoral pseudoaneurysm and concurrent metastatic septic arthritis in a high-risk hemodialysis patient. This approach challenges the traditional paradigm of mandatory surgical intervention for these severe complications and offers a potential therapeutic alternative for carefully selected patients deemed poor surgical candidates. Future studies, such as registries or multicenter case series, are needed to better define the clinical, microbiological, and radiological parameters that predict successful outcomes with conservative management in this challenging patient population.
